# Genome-Wide Analysis of the *Nramp* Gene Family in Kenaf (*Hibiscus cannabinus*): Identification, Expression Analysis, and Response to Cadmium Stress

**DOI:** 10.3390/plants13172514

**Published:** 2024-09-07

**Authors:** Qin Liu, Shaocui Li, Guanghui Du, Xia An

**Affiliations:** 1 School of Agriculture, Yunnan University, Kunming 650500, China; lq18388746921@163.com; 2 Zhejiang Xiaoshan Institute of Cotton&Bast Fiber Crops, Zhejiang Institute of Landscape Plants and Flowers, Zhejiang Academy of Agricultural Sciences, Hangzhou 311251, China; lishaocui1992@163.com

**Keywords:** *Hibiscus cannabinus*, *Nramp* gene family, cadmium, phylogenetic analysis

## Abstract

Kenaf (*Hibiscus cannabinu*) is a grass bast fiber crop that has the ability to tolerate and accumulate heavy metals, and it has been considered as a potential heavy metal accumulator and remediation plant. Nramp is a natural resistance-related macrophage, which plays an important role in the transport of divalent metal ions, plant growth and development, and abiotic stress. In this study, the *Nramp* gene family of kenaf was analyzed at the whole genome level. A total of 15 *HcNramp* genes were identified. They are distributed unevenly on chromosomes. Phylogenetic analysis classified 15 HcNramp proteins into 3 different subfamilies. All proteins share specific motif 4 and motif 6, and the genes belonging to the same subfamily are similar in structure and motif. The promoters are rich in hormone response, meristem expression, and environmental stress response elements. Under different treatments, the expression levels of *HcNramp* genes vary in different tissues, and most of them are expressed in roots first. These findings can provide a basis for understanding the potential role of the *Nramp* gene family in kenaf in response to cadmium (Cd) stress, and are of great significance for screening related Cd tolerance genes in kenaf.

## 1. Introduction

Heavy metal pollution is one of the most serious environmental problems, with cadmium (Cd) pollution being a primary concern among heavy metal pollutants. Cd stress significantly impacts plant growth and development, altering various physiological and metabolic processes [1,2,3,4]. Unlike other elements, Cd does not have a dedicated transporter in plants. It enters the roots and is transported to the shoots by competing with other nutrients for transport pathways [5]. Cd stress can trigger the accumulation of reactive oxygen species in plants, leading to decreased activities of superoxide dismutase (SOD) and peroxidase (POD) enzymes, thereby reducing antioxidant and detoxification capacities. In turn, it disrupts the antioxidant system and can cause membrane lipid peroxidation [1,4]. Hyperaccumulator plants have efficient Cd absorption, transportation, and detoxification systems, and are the main materials for remediation of heavy-metal-contaminated soil, which can be used to study the adaptability of plants in extreme environments [6].

Kenaf (*Hibiscus cannabinus* L.), also known as hibiscus, is an annual herbaceous bast fiber crop in the Malvaceae [7]. It boasts a short growth cycle coupled with substantial biomass yield [8,9]. Kenaf and other bast fiber crops possess the characteristics of wide cultivation adaptability, making them versatile and multifunctional. Kenaf is recognized as a new type of papermaking raw material that can replace wood pulp. Additionally, it serves as an important raw material in the traditional bast fiber textile industry, and has diverse applications, such as automobile lining, paper mulching, fluff pulp, sewage purification material, soil conditioner, plastic filler, activated carbon, and environmental protection adsorption material, and is known as a “potential superior crop in the 21st century” and “futuristic crop” [6,8,9,10,11]. Kenaf exhibits strong heavy metal tolerance and accumulation ability [1], and is not involved in the circulation of the food chain. It is considered to be a potential plant for repairing heavy metal pollution [1,12]. Cultivating crops in soils contaminated with heavy metals facilitates the remediation of degraded agricultural lands and enables the productive utilization of otherwise uncultivated areas [6,12]. In the treatment of heavy metal Cd stress, adding different forms and concentrations of nitrogen fertilizer can promote the growth of kenaf [13]. These crops can simultaneously provide fiber biomass raw materials for both traditional and innovative industries, offering new avenues for agricultural and industrial chain innovation [1].

Nramp (nature resistance-associated macrophage protein) is a natural resistance-associated macrophage protein [14]. It is an important proton/metal transporter in plants [15,16] and participates in the transport of heavy metals, such as zinc (Zn), iron (Fe), manganese (Mn), Cd, and arsenic (As) [5,17,18,19,20,21,22,23]. At the same time, it plays an important role in the transmembrane transport of divalent metals [5,10,15]. The Nramp sequence has a similar secondary structure among species [18], with 10–12 highly conserved transmembrane domains (TMD) [16,18,22,24], and has the structural characteristics of ion channels and ion transporters in evolution [18]. The gene family plays an important regulatory role in maintaining cell homeostasis, photosynthesis, protein activity, and plant response to environmental stress [18,24,25]. It keeps the balance of metal ions in plants through the transport of metal ions. It plays an important role in the detoxification of heavy metals [18,26,27] and has ion-selective changes in different genes [24]. Nramp proteins have been discovered in a variety of plants, including *Arabidopsis thaliana* [28], tomato (*Solanum lycopersicum*) [21], seabuckthorn (*Hippophae rhamnoides*) [25], soybean (*Glycine max*) [20], rice (*Oryza sativa*) [29], *Populus trichocarpa* [14], and potato (*Solanum tuberosum*) [21]. Nramp protein is the key to plant uptake and transport of Cd [6].

There is a lot of evidence that *Nramp* transcription factors in plants play a key role in various biological processes. *AtNramp1* is a transporter encoding Fe, Mn, and Cd [21,23,30], which is a key transporter of Mn absorption under low Mn conditions [18]. In response to Cd treatment, *AtNramp3* undergoes overexpression, thereby assuming a pivotal role in facilitating Fe transport and modulating the sensitivity of plants to Cd [28]. *AtNramp6* functions as a transporter for both Cd and Fe [23], exhibiting a dual role in nutrient and metal homeostasis. When Fe is deficient, *AtNramp6* has been shown to influence the growth of lateral roots in mustard plants [30], suggesting its importance in adapting to Fe limiting conditions. Despite its high sensitivity to Cd, *AtNramp6* does not significantly alter the overall Cd content within plants. Rather, it appears to enhance the tolerance of plants to Cd stress, allowing them to better withstand exposure to this toxic metal [18,25,30]. Rice *OsNramp1* is a transporter that plays a pivotal role in the absorption and translocation of Cd. Its involvement promotes the accumulation of both As and Cd within the plant. Specifically, when *OsNramp1* is overexpressed, it enhances the accumulation of Cd in leaves, contributing to elevated Cd levels in the photosynthetic tissues. Furthermore, when overexpressed in roots, *OsNramp1* increases the translocation of Cd to stems, indicating its role in facilitating the long-distance movement of this toxic metal within the rice plant [18]. *OsNramp6* is a Fe and Mn transporter located in the plasma membrane, which is helpful for disease resistance [5,19]. Tomato *SlNramp2* and *SlNramp3* are genes related to Cd absorption by roots, and they are highly expressed under Cd stress [31]. *SlNramp3* is a heavy metal transporter, which is involved in the transport of heavy metals from roots to stems [32]. *SaNramp1* from *Sedum alfredii* can transport Cd, Mn, and Zn, and its overexpression in tobacco significantly increases the contents of Cd, Zn, and Mn above ground [33]. Overexpression of *TtNramp1* can improve Cd transport, while overexpression of *TtNramp6* can increase Cd content in plants and promote Cd accumulation [34]. Tian et al. identified and analyzed the *Nramp* gene family of potato and its response to five kinds of heavy metal stresses, such as Cu^2+^, Cd^2+^, and Zn^2+^ [21]. Chen et al. identified the whole genome of the *Nramp* gene family of *Spirodela polyrhiza* and analyzed the expression of Cd stress [19]. Hussain et al. identified and analyzed the whole genome of the *Nramp* gene family in *Kandelia obovate* and its response to copper (Cu) stress [35]. Genome-wide identification and evolutionary analysis of the *Nramp* gene family in the AC genome of *Brassica* species was performed by Zhao et al. [27]. However, there is no systematic analysis of *Nramp* transcription factors in kenaf to clarify their role in responding to heavy metal stress.

In this study, the *Nramp* of kenaf was comprehensively analyzed. Fifteen *Nramp* genes were identified by bioinformatics, and the phylogenetic analysis, conserved motifs, domains, gene structure, subcellular localization, chromosome position, cis-regulatory elements, and expression profile were deeply analyzed at the whole genome level. In addition, qRT-PCR was used to detect the effects of four treatments on the expression pattern of *HcNramps*. Our findings serve as a solid foundation for future endeavors aimed at unraveling the multifaceted functions of the *Nramp* gene family in kenaf’s resilience against heavy metal stress. They offer invaluable insights into the evolutionary mechanisms governing *HcNramps*, while simultaneously presenting novel strategies to tackle the challenge of heavy metal accumulation in plants. In essence, this study provides insights into a deeper understanding of the interactions between plants and metals and the development of innovative solutions to reduce environmental pollution.

## 2. Results

### 2.1. Identification and Characterization of Nramp Genes in Kenaf

Following a search of the kenaf genome database using HMMER 3.0, the identified candidate sequences were examined using NCBI Batch CDD, Pfam, and SMART to confirm the presence of the Nramp domain. A total of 15 *HcNramp* gene family members were identified from the kenaf genome (Table S1), which were systematically named based on their chromosomal positions, designated as *HcNramp1* through *HcNramp15* (Table S2). The analysis of the physicochemical properties revealed a diverse range of characteristics among the identified proteins. Specifically, the length varied from 221 to 1379 amino acids, with the relative molecular weight range from 24,223.94 to 150,763.33 Da. The theoretical isoelectric point (pI) ranged from 5.08 to 10.18, with one member having a pI value of 7. Seven were alkaline proteins and another eight were acidic proteins. The instability coefficient ranged from 30.06 to 44.88. Among them, HcNramp2/8/9/10/12 protein was unstable. From the Aliphatic index, HcNramp7 protein was the most stable, with a reading of 124.21. HcNramp12 protein was the most unstable, with a reading of 89.04. The average total hydrophilicity (GRAVY) ranged from −0.092 to 0.656, and HcNramp2/9/10/12 were hydrophilic proteins, and the remaining proteins were hydrophobic proteins. Subcellular localization showed that all HcNramp proteins were located on the plasma membrane, HcNramp3/5 proteins were also located in the vacuole, and HcNramp11 proteins were located on the endoplasmic reticulum.

### 2.2. Secondary Structure Analysis of HcNramp Family Proteins

The secondary structures of HcNramp family proteins were mainly composed of α-helix, extended chain, folding, and random coil (Table S3). Among them, the proportion of α-helix was the highest, ranging from 39.85% to 59.08%, and the proportion of extended chain was the least, ranging from 5.15% to 13.57%. Only HcNramp2 protein had folding, no random coil, and the rest of the proteins had random coil, indicating that the protein structures were stable. The proportion of secondary structures of HcNramp8/9/10 was extended strand < alpha-helix < random coil, which indicates that the three protein structures were variable and may be the sites of proteins with special functions.

### 2.3. Classification, Phylogenetic Analysis, Gene Structure, and Conserved Motifs of the HcNramp Gene Family

**Figure 1:**
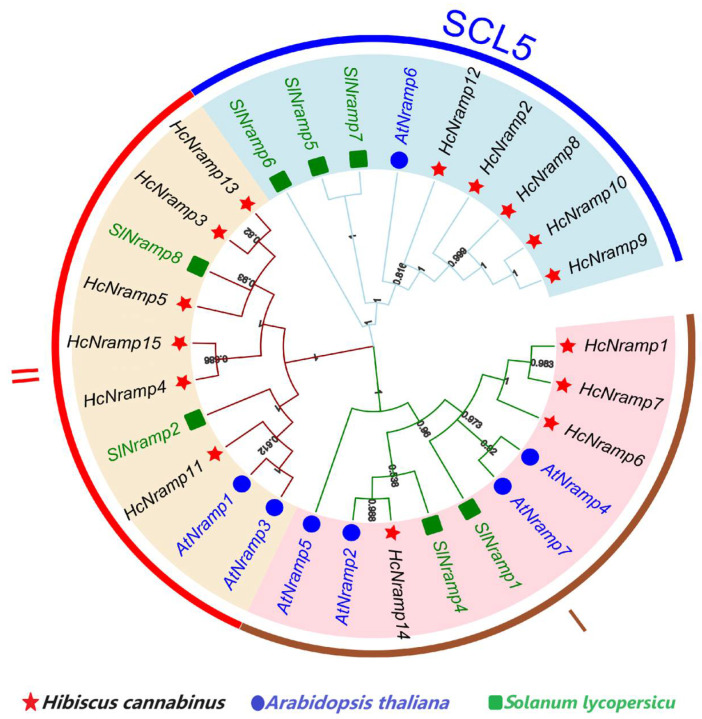
Phylogenetic analysis of 29 Nramp proteins in kenaf, *A. thaliana*, and tomato. Fifteen proteins of kenaf are labeled in black font, seven proteins of *A. thaliana* are in blue font, and seven proteins of tomato are in green font. The maximum likelihood tree was created using MEGAX (bootstrap value = 1000) and the bootstrap value of each branch is displayed.

In order to understand the evolutionary relationship of the *Nramp* gene family in kenaf and other species, a phylogenetic tree of 29 Nramp protein sequences was constructed, including 15 HcNramp kenaf proteins, 7 *A. thaliana* proteins, and 7 tomato proteins (Table S4). These three species were divided into three categories (Figure 1), which were the I, II, and SLC5 subfamilies. HcNramp1/6/7/14 belong to subfamily I, with proteins ranging in length from 501 to 564 amino acids, theoretical pI ranging from 5.08 to 5.41, instability coefficient ranging from 32.56 to 37.38, and Aliphatic index ranging from 116.17 to 124.21, indicating that the proteins in this subfamily were stable, all of which were hydrophobic proteins, and this subfamily was secondary in protein. HcNramp3/4/5/11/13/15 belong to subfamily II, with amino acids ranging from 221 to 649, pI ranging from 7.12 to 10.18, instability coefficient ranging from 30.06 to 33.82, and Aliphatic index ranging from 122.53 to 106.97. The proteins were stable and all hydrophobic. HcNramp2/8/9/10/12 belong to the SCL5 subfamily, with the proteins ranging in length from 1300 to 1379 amino acids, the theoretical pI ranging from 5.43 to 7, the instability coefficient ranging from 41.11 to 44.88, and the Aliphatic index ranging from 89.04 to 94.10. All proteins were unstable except HcNramp in the SCL5 subfamily. In the secondary structure of proteins, the proportion of folding was the highest in HcNramp2, and the proportion of random curl was the highest in HcNramp8/9/10. The proportion of α-helix and random curl in HcNramp12 is the same. Protein in the same subfamily is species-specific, indicating that these proteins may have similar structures. The cross-species phylogenetic analysis of Nramps in kenaf, *A. thaliana*, and tomato showed that all the homologous Nramps could be well clustered together (Figure 1). HcNramp1/6/7/14 in kenaf, AtNramp2/5/4/7 in *A. thaliana*, and SlNramp1/4 in tomato belong to subfamily I. HcNramp3/4/5/11/13/15 in kenaf, AtNramp1/3 in *A. thaliana*, and SlNramp2/8 in tomato belong to subfamily II. HcNramp2/8/9/10/12 in kenaf, AtNramp6 in *A. thaliana*, and SlNramp5/6/7 in tomato belong to subfamily SCL5.

**Figure 2:**
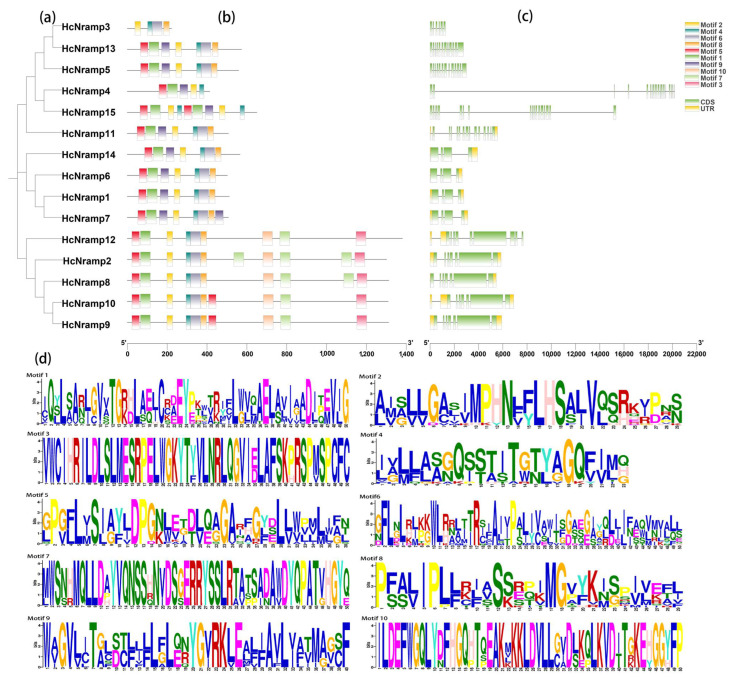
Conservation motif and gene structure analysis of *HcNramp* genes according to the phylogenetic relationship. (**a**) The phylogenetic relationship of HcNramps. (**b**) Conserved motifs of HcNramps. Different colors represent different motifs. (**c**) Exon and intron structures of the *HcNramp* genes in kenaf. The grey lines indicate introns, the green boxes represent exons, and the orange boxes indicate untranslated regions. (**d**) The amino acid composition of each motif.

The conserved motif prediction of the kenaf Nramp protein sequence was performed using MEME Suite 5.5.5 online software. The results showed that the motifs between different subfamilies were different, and the motifs between members of the same subfamily were relatively conserved (Figure 2 and Table S5). In the Nramp gene family of kenaf, all 15 *HcNramps* contained motif 4 and motif 6, indicating that the motif of the kenaf Nramp gene is relatively conservative. Most of the HcNramps contained motifs 1/2/4/5/6/8/9, where only *HcNramp4* and *HcNramp5* contained only motifs 4 and 5, respectively. The difference in the number of conserved motifs of the gene indicates that the gene has functional differences with other genes and may have different biochemical characteristics and biological functions [3]. Gene structure analysis showed that each gene contained introns, and the number of introns varied greatly, with a maximum of 17 introns and a minimum of 3 introns. The *HcNramp* genes clustered in the same branch of the phylogenetic tree were similar in exon–intron tissues. The *HcNramp3/4/5/13/15* genes in the same branch did not contain untranslated regions (UTRs) at the 5’ and 3’ ends. Among them, *HcNramp12* did not contain untranslated regions (UTRs) at the 3’ end, and the remaining nine genes had complete gene structures.

### 2.4. Analysis of Cis-Acting Elements of HcNramp Promoter

**Figure 3:**
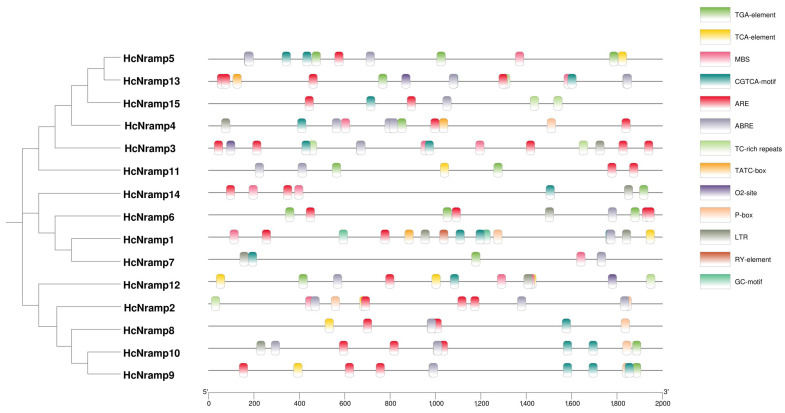
Identification of the cis-acting elements in the promoter of *HcNramp genes*.

The cis-regulatory elements in the promoter region (including 2,000 base pairs upstream) were comprehensively analyzed and screened, and the online software PlantCare was used for analysis (Figure 3). The results showed that 156 cis-acting elements were divided into 3 parts, including abiotic stress response elements (67 elements accounted for 42.95%), hormone response elements (85 elements accounted for 54.49%), and growth and development response elements (4 elements accounted for 2.56%). *HcNramp* gene was involved in a complex regulatory network and was regulated by various environmental, developmental, and physiological factors.

The abiotic stress elements are composed of hypoxia/anaerobic induction, defense and stress elements, and cis-elements necessary for drought and low temperature. These abiotic stresses were composed of 2 GC-motifs (2.99%), 37 AREs (55.22%), 7 TC-rich repeats (10.45%), 12 MBSs (17.91%), and 9 LTRs (13.43%). *HcNramp3* contains ten abiotic stress response elements, including two TC-rich repeats, two MBSs, one LTR, and five AREs; secondly, *HcNramp1* contains seven abiotic stress response elements, including one MBS, two LTRs, two GC-motifs, and two AREs. This indicates that *HcNramp3* may play an important role in anaerobic stress.

The hormone response elements include auxin, gibberellin, salicylic acid, abscisic acid, and methyl jasmonate response elements. They were composed of 15 TGA elements (17.65%), 3 TATC-box (3.53%), 7 P-box (8.24%), 9 TCA elements (10.59%), 31 ABREs (36.47%), and 20 CGTCA motifs (23.53%). *HcNramp5* has 10 hormone response elements, including 3 TGA elements, 1 TCA element, 2 CGTCA motifs, and 4 ABREs, indicating that the gene may be involved in the regulation of abscisic acid. Secondly, there were two genes containing eight hormone response elements, namely, *HcNramp1* (including one TCA element, one TATC-box, one p-box, three CGTCA motifs, and two ABREs) and *HcNramp9* (one TGA element, one TCA element, one P-box, three CGTCA motifs, and two ABREs), indicating that *HcNramp1* and *HcNramp9* genes are involved in the regulation of methyl jasmonate.

Growth and development response elements, including seed-specific cis-acting elements and zein metabolic regulation, were composed of one RY element (25%) and three O2 sites (75%). Among them, *HcNramp1* contains a RY element, which indicates that the gene is involved in the specific regulation of seed expression. *HcNramp3/2/13* all contained one O2 site, indicating that these three genes may be involved in the metabolism of gliadin.

### 2.5. Chromosomal Localization and Collinearity Analysis of HcNramp Genes

**Figure 4:**
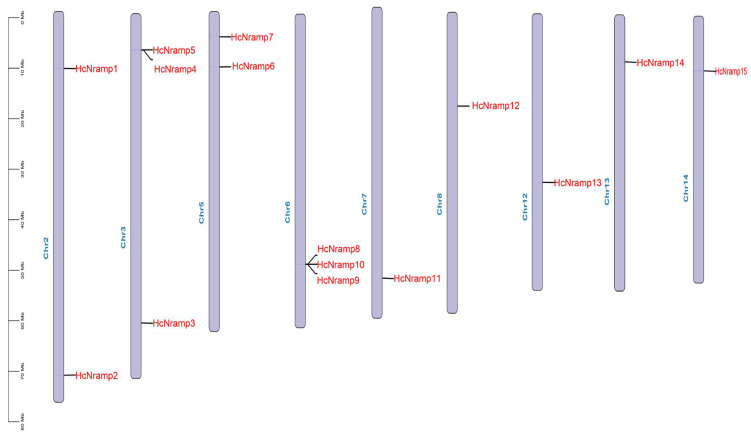
Chromosome location of *HcNramp* genes. Chromosome numbers are shown to the left of the chromosomes. *Nramp* genes are labeled to the right of the chromosomes. Scale bar on the left indicates the chromosome lengths (Mb).

The 15 *HcNramp* genes were located on 9 chromosomes, and the distribution of each gene on the chromosome was uneven. The *HcNramp* gene was most distributed on Chr3 and Chr6 chromosomes, with three genes located, followed by two genes located on both Chr2 and Chr5 chromosomes, and only one gene located on Chr7, Chr8, Chr12, Chr13, and Chr14 (Figure 4).

**Figure 5:**
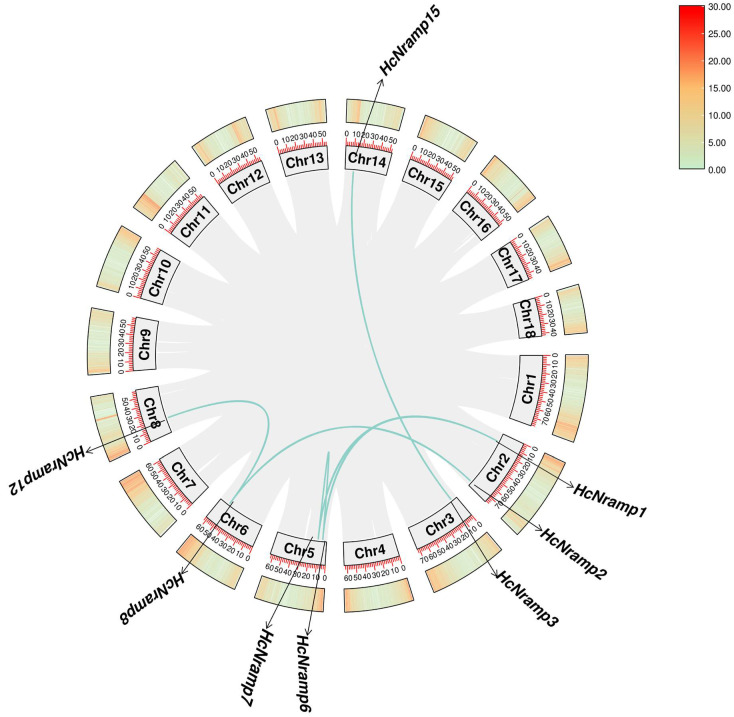
Intraspecific collinearity analysis of the kenaf *Nramp* gene family. The green lines in the figure are common genes, and the gray lines represent collinear blocks of the plant genome.

**Figure 6:**
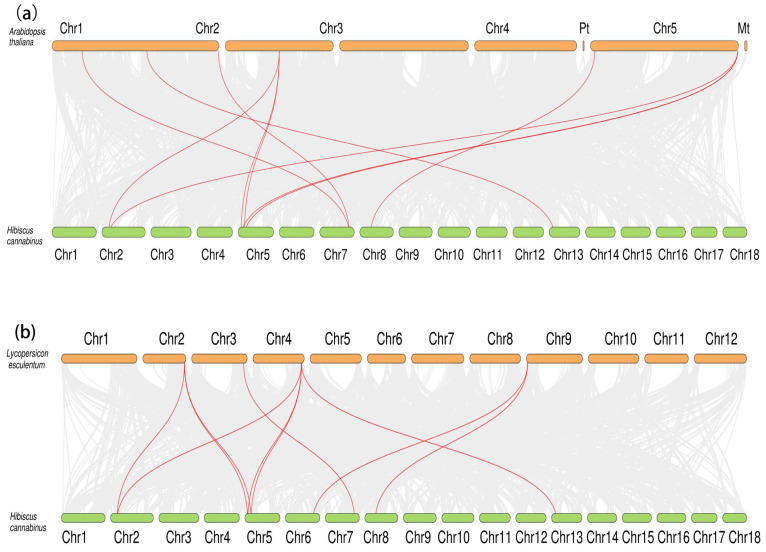
Interspecific collinearity analysis of the kenaf *Nramp* gene family. (**a**) Interspecific collinearity analysis of kenaf and *A. thaliana*. (**b**) Interspecific collinearity analysis of kenaf and tomato. The red lines in the figure are the common genes between kenaf, *A. thaliana,* and tomato, and the gray lines represent the collinear blocks of the plant genome.

In order to clarify the evolutionary relationship, the replication of the *HcNramp* gene was studied. It was found that there were six collinear gene pairs in the kenaf species (Figure 5), including H*cNramp1:HcNramp6*, *HcNramp1*:*HcNramp7*, and *HcNramp6*:*HcNramp7* in subfamily I, *HcNramp3*:*HcNramp15* in subfamily II, and *HcNramp2*:*HcNramp8* in subfamily SCL5. In order to further study the homology of *Nramp* gene members among different species, collinear maps of *Nramp* gene families of kenaf and *A. thaliana*, and kenaf and tomato, were drawn. There were ten collinear gene pairs in kenaf and *A. thaliana*, and ten collinear gene pairs in kenaf and tomato (Figure 6 and Table S6).

### 2.6. Tissue-Specific Expression Analysis of the HcNramp Genes

**Figure 7:**
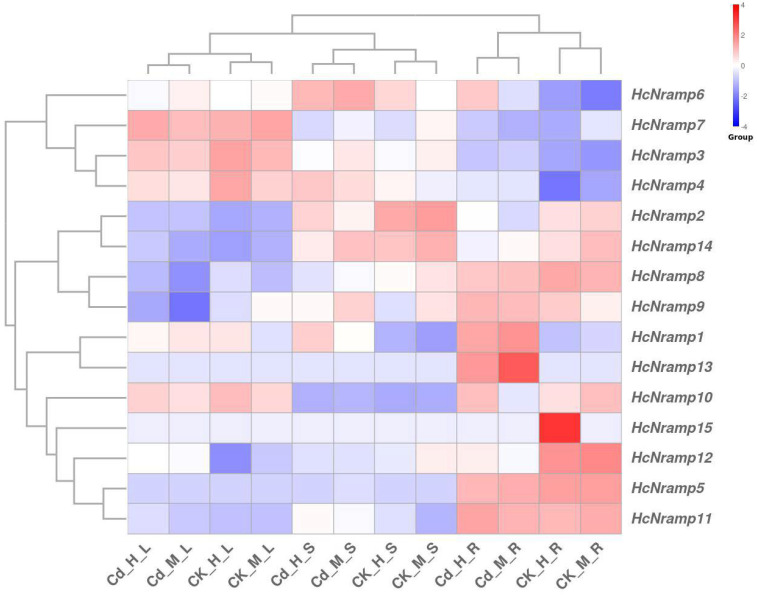
Tissue-specific expression profiles of *HcNramp* genes in kenaf. CK-H-R represents the root samples treated by CK + water spraying, CK-M-R represents the root samples treated by CK + melatonin spraying, Cd-H-R represents the root samples treated by Cd + water spraying, and Cd-M-R represents the root samples treated by Cd + melatonin spraying. L, S, and R represent the leaves, stems, and roots of kenaf samples. Red and blue boxes indicate high and low expression levels of genes, respectively.

The expression pattern of a gene is crucial for elucidating its function [27]. In order to further explore the role of *HcNramp* genes, a heat map showing the expression pattern of the *HcNramp* gene was constructed by using the transcriptome data (Accession: PRJNA1134624). Tissue-specific expression analysis was carried out by calculating the TPM value of the gene. All *HcNramp* genes showed tissue-specific expression (Figure 7 and Table S7). Fifteen *HcNramp* genes were divided into three different groups: the first group was *HcNramp3/4/6/7*, the second group was *HcNramp2/8/9/14*, and the third group was *HcNramp1/5/10/11/12/13/15*. The gene expression levels were different in different treatments, with significant differences between normal treatment and Cd treatment. The expression trends of genes in CK-H and CK-M, and Cd-H and Cd-M treatments were similar, but the specific expression levels were different. *HcNramp1*/*6* were expressed relatively high in Cd treatment. *HcNramp2/14* was highly expressed in the roots and stems of CK-H and CK-M treatments. *HcNramp7* was preferentially expressed in leaves, *HcNramp2/6/14* in stems, and *HcNramp1/5/8/9/11/12/13/15* in roots. *HcNramp3/4/7* was low expressed in roots and *HcNramp10* in stems. *HcNramp2/14/8/9/11* was low or not expressed in leaves, while *HcNramp5/13/15* was low expressed in stems and leaves. *HcNramp13* was highly expressed in Cd-M-R, and *HcNramp15* was highly expressed in CK-H-R. *HcNramp4* was low expressed in CK-H-R, *HcNramp6* was low expressed in CK-M-R, *HcNramp9* was low expressed in Cd-M-L, and *HcNramp12* was low expressed in CK-H-L.

### 2.7. HcNramp Gene Expressions under Exposure to Different Treatments of Heavy Metals

**Figure 8:**
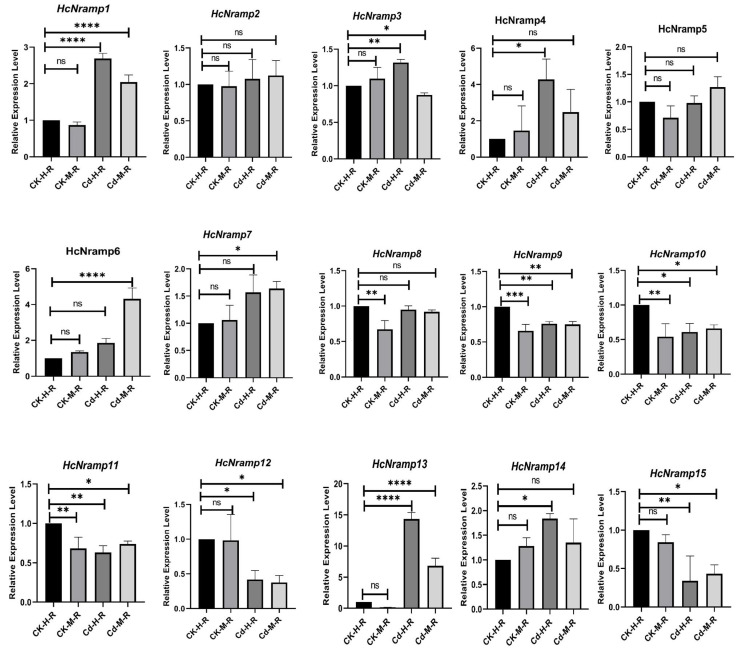
The expression levels of *HcNramp* genes in kenaf roots under different treatments. Data represent means ± SD of three biological replicates. CK-H-R represents control samples. The expression levels of each gene were normalized against its own expression level in the root tissue in control (CK-H-R) conditions. Error bars indicate mean ± SD and asterisks indicate statistical differences between the treatment samples and the corresponding control samples, the roots (n = 3; * *p* < 0.05, ** *p* < 0.01, and *** *p* < 0.001, **** *p* < 0.0001 and ns not significant; Student’s *t*-test).

**Figure 9:**
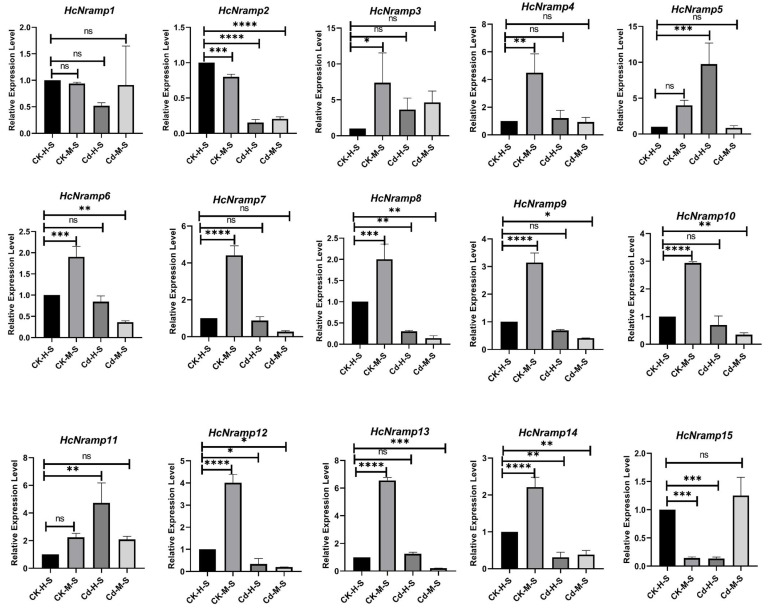
The expression levels of *HcNramp* genes in kenaf stems under different treatments. Data represent means ± SD of three biological replicates. CK-H-S represents control samples. The expression levels of each gene were normalized against its own expression level in the stem tissue in control (CK-H-S) conditions. Error bars indicate mean ± SD and asterisks indicate statistical differences between the treatment samples and the corresponding control samples, the stems (n = 3; * *p* < 0.05, ** *p* < 0.01, *** *p* < 0.001, **** *p* < 0.0001 and ns not significant; Student’s *t*-test).

**Figure 10:**
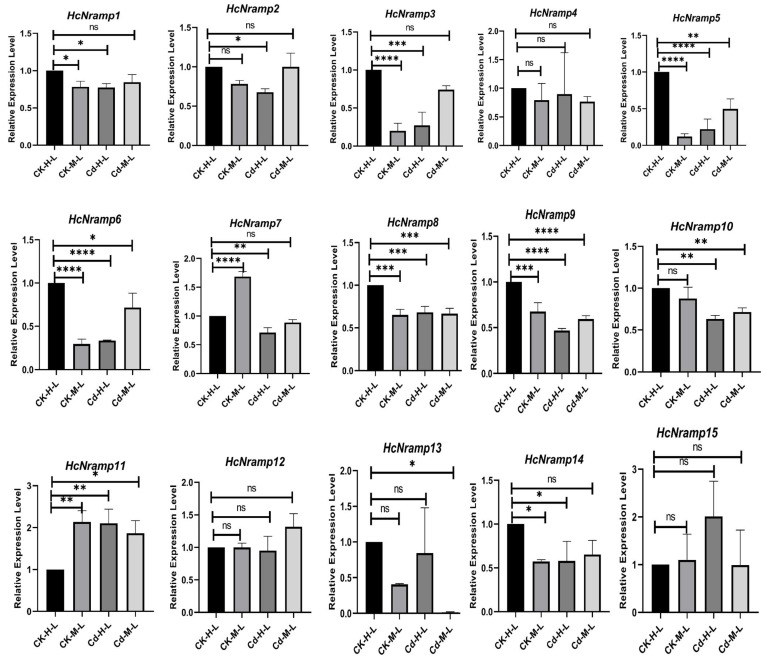
The expression levels of *HcNramp* genes in kenaf leaves under different treatments. Data represent means ± SD of three biological replicates. CK-H-L represents control samples. The expression levels of each gene were normalized against its own expression level in the leaf tissue in control (CK-H-L) conditions. Error bars indicate mean ± SD and asterisks indicate statistical differences between the treatment samples and the corresponding control samples, the leaves (n = 3; * *p* < 0.05, ** *p* < 0.01, and *** *p* < 0.001, **** *p* < 0.0001 and ns not significant; Student’s *t*-test).

To clarify the response of the *HcNramp* genes to Cd stress, we used qRT-PCR to analyze the gene expression level in roots, stems, and leaves under four different treatment conditions (Figure 8, Figure 9 and Figure 10). There were notable differences in the expression patterns of *HcNramp* genes across roots, stems, and leaves, all significantly influenced by Cd stress. According to the results of the relative gene expression levels in kenaf roots (Figure 8), Cd treatment alone significantly upregulated the expression of *HcNramp1/4/7/13/14* compared to the control. Furthermore, combined treatment of Cd and melatonin significantly enhanced the expression of HcNramp1/4/6/7/13, but the expression levels of other genes did not change significantly. In the kenaf stems (Figure 9), melatonin treatment alone significantly increased the expression of HcNramp3/4/5/6/7/8/9/10/11/12/13/14 compared to the control. On the other hand, Cd treatment significantly upregulated the expression of HcNramp3/5/11. In the kenaf leaves (Figure 10), melatonin treatment significantly increased the expression of HcNramp7 and 11 compared to the control. Cd treatment alone significantly upregulated the expression of HcNramp11/15, while combined Cd and melatonin treatment significantly enhanced the expression of *HcNramp11*. These findings provide valuable insights into the differential regulation of *HcNramp* genes in response to Cd stress and melatonin treatment, highlighting their potential roles in Cd tolerance and detoxification mechanisms in kenaf.

## 3. Discussion

Kenaf is an annual herbaceous fiber crop, which has the characteristics of a short growth cycle, large biomass, and not participating in the circulation of the food chain [6]. It can not only be used to remediate polluted soil, but also has certain economic benefits. Kenaf is considered as a potential plant to remediate soil contaminated by heavy metals and has attracted much attention in the field of heavy metals [8,10]. Therefore, it is of great significance to analyze the *Nramp* gene family of kenaf using bioinformatics.

The *Nramp* gene plays an important role in the absorption and transport of metal ions. A large number of *Nramp* genes have been identified functionally [34] and have been analyzed in many species [24]. In our study, fifteen members of the *Nramp* gene family were identified from the whole genome of kenaf. Compared with *A. thaliana* (7) and tomato (9) [21], the number of *Nramp* in kenaf was the largest. The subcellular localization of the *Nramp* gene family in kenaf is mostly located in the plasma membrane, and a few are located in the vacuole membrane and endoplasmic reticulum. Protein located in the plasma membrane mainly absorbs metal ions from plants in vitro, which may be related to its function of transporting heavy metal ions across membranes [23]. Protein located in the vacuole membrane is responsible for transporting metal ions and their steady state [25]. Protein located in endoplasmic reticulum is mainly involved in the process of cell synthesis and modification of protein. Therefore, it can be inferred that kenaf is a heavy-metal-rich plant with a strong absorption ability and weak transport ability.

Phylogenetic analysis clustered the Nramp proteins into three distinct subfamilies, which was consistent with the assessment that subdivided *A. thaliana* Nramp proteins into three subgroups (Figure 1) [21]. According to previous studies, different structures and subfamilies have different functions [7]. Further *HcNramp* gene structure analysis (Figure 2) showed that the members of the most closely related subfamilies usually shared similar exon–intron structures, indicating they have similar evolutionary relationships. All members of the *HcNramp* gene family contained introns, and conserved motifs 4 and 6, and only the protein in the SCL5 subfamily had motif 3. All members contained motif 4 (GQSSTITGTYAGQFIMQ), a transport motif of the *Nramp* family, which indicated that the *Nramp* family is highly conserved [25]. Furthermore, the *HcNramp* genes in the different subfamilies displayed different exon–intron structures, while subtle differences may play a critical role in the gene evolution progress. Therefore, it is speculated that different gene structures are involved in divergent functions.

Twenty-nine protein sequences of kenaf, *A. thaliana*, and tomato were used to construct the phylogenetic tree. According to the phylogenetic tree, they can be divided into three subfamilies. According to the grouping of *A. thaliana* and tomato, these three subfamilies were named I, II, and SCL5, respectively [21]. The protein grouping of *A. thaliana* and tomato in each branch of the phylogenetic tree was consistent with Tian’s findings [21]. Different species have different evolutionary speeds, so there are differences in the classification of evolutionary trees. The distribution of *HcNramp* genes in the three subfamilies of kenaf was uneven. Subfamily I contained four *HcNramp* genes, subfamily II contained six *HcNramp* genes, and subfamily SCL5 contained five *HcNramp* genes. These differences indicate that there is asymmetric evolution among the three subfamilies of kenaf [5]. In this study, *SlNramp3* and *SlNramp9* proteins were not mentioned because *SlNramp3* and *SlNramp9* in tomato belong to the Mnth family, while *HcNramp* in kenaf has only three subfamilies, and there are no members of the Mnth family in *A. thaliana* and kenaf [21]. The *Nramps* of *A. thaliana* and tomato were collinear with 15 *HcNramp* genes of kenaf, and there were 10 collinear gene pairs between the 2 species and kenaf, which indicated that there was a close evolutionary relationship between them. Among them, *HcNramp1/6/7/11/12/14* genes of kenaf had collinear gene pairs with *AtNramp1/2/3/4/6/7* of *A. thaliana*, and *HcNramp1/2/6/7/8/11/12/14* had collinear gene pairs with *SlNramp1/2/3/7* of tomato. Previous studies have mentioned that there are multiple collinear gene pairs between species, which indicates that there are multiple gene copies in the process of species evolution [36]. There were 6 pairs of collinear genes in kenaf, 10 pairs in kenaf and *A. thaliana*, and 10 pairs in kenaf and tomato. Previous studies have mentioned that there are multiple collinear gene pairs between species, which indicated that there are multiple gene copies in species evolution [21].

The *Nramp* family is a family of transporters, which can transport divalent metal cations. All *HcNramp* have transport motifs [25], so it is speculated that these proteins may transport Cd related to transport motifs. The cis-acting promoter elements were identified to address *HcNramp* gene expression regulation. The analysis of cis-acting elements of promoters showed that the promoter sequences of genes in the same subfamily were similar. The promoter region of *HcNramps* contained a variety of stress response elements and hormone response elements, so it is speculated that *HcNramps* genes may be regulated by complex regulatory networks [21]. Studies have shown that some phytohormones, such as jasmonic acid (JA) and SA, participate in the plant response to different metal stress [37,38]. We also found that in the *HcNramp* gene promoters, there were many cis elements associated with heavy metal stress, which may be involved in the transport and absorption of metals. For example, SA significantly reduces Cd in rice grains by regulating the expression levels of *OsNramp2* associated with Cd translocation and accumulation [39]. Among the various *HcNramp* genes, *HcNramp2/3/12/13/15* are unique in possessing TC-rich repetitive sequences, which may confer specific regulatory or structural properties.

The expression of 15 *HcNramp* genes was divided into 3 different groups. The expression trends of the genes in the same group were similar, with significant differences between the different groups [24]. In different treatments, the expression level of the genes was different, and there was a significant difference between CK and Cd treatments. However, the expression trends of CK-H and CK-M, and Cd-H and Cd-M, were similar, but the specific expression levels were different. It is speculated that the reason for the above situation may be that the *Nramp* gene family is mainly involved in the transport of divalent cations, and *HcNramps* may be involved in the transport of Cd^2+^, so there is a significant difference in expression between CK and Cd treatments, and the expressions in CK-H and CK-M, and Cd-H and Cd-M, are similar. There are significant differences between CK and Cd treatments in *BnNRAMPs* (*Brassica napus*) [27], between CK and Pb (lead) stress in *HrLNRAMPs* [25], and between CK and Cu treatments in *KoNRAMPs* (*Kandelia obovata*) [35], which are consistent with the significant differences in the expression of *HcNramps* between CK and Cd treatments in this paper. These results were consistent with previous studies, indicating that *Nramps* were responsible for Cd uptake and transport.

*HcNramp3* exhibited preferential expression in leaves, whereas the remaining four genes, *HcNramp2/12/13/15*, were predominantly expressed in roots. This differential expression pattern suggests that these genes may play an important role in roots, as they may be induced by stress. This function is similar to the mechanisms observed in peanuts [24]. *AtNramp1* is a Cd transporter [21,30], and *AtNramp3* plays a role in Cd sensitivity [28]. *HcNramp3/11* are homologous genes, so it can be speculated that these genes may be candidates for Cd regulation of kenaf. *AtNramp5* is involved in Cd transport [19], and it has homology with four *HcNramp5* genes in subfamily I, and all of them are located in stable hydrophobic proteins on the plasma membrane, so it is speculated that *HcNramp1/6/7/14* genes may be involved in Cd transport. *AtNramp6* is a transport protein of Cd [23], and it can also improve the tolerance of plants to heavy metal Cd [18,25,30]. It has homology with five genes of the SCL5 subfamily, indicating that *HcNramp2/6/8/9/12* may also improve the tolerance of kenaf to Cd. The tolerance of heavy metal hyperaccumulator plants and their ability to accumulate metals mainly depend on their adaptation to the dynamic balance of metals. In some hyperaccumulator plants, the *Nramp* metal transporter is highly expressed [40]. High-accumulation plants can enrich toxic heavy metals through the *Nramp* gene, which can improve the soil where heavy metals accumulate and is beneficial to the development of phytoremediation biotechnology of transgenic plants [18]. Therefore, the *Nramp* gene family can be excavated more comprehensively and systematically, and its biological function can be deeply analyzed.

## 4. Materials and Methods

### 4.1. Identification and Sequence Analysis of Nramp Genes in Kenaf

The genomic data of kenaf were obtained from the nucleic acid sequence archiving system (CNCB; https://download.cncb.ac.cn/gwh/Plants/Hibiscus_cannabinus_Fuhong952_GWHACDB00000000.1/, accessed on 6 May 2024) of the China National Center for Bioinformation [41], including genome-wide files, protein sequence files, and GFF annotation files. The genomic files of *A. thaliana* and tomato were download from EnsembPlants (https://ftp.Ensemblgenomes.ebi.ac.uk/pub/plants/release-59/, accessed on 16 May 2024). The protein sequence files of kenaf, *A. thaliana*, and tomato were obtained by TBtools-II (Toolbox for Biologists) v2.119 software [42,43]. The Hidden Markov Model (HMM) profile of the Nramp domain (PF01566) was obtained from the Pfam database (https://pfam.xfam.org/, accessed on 10 May 2024) [20], and we used TBtools software [42,43] to localize Blast to obtain the protein sequences of members of the *Nramp* gene family. We obtain the candidate proteins of kenaf, *A. thaliana*, and tomato by Hmm search with an E value of <0.001, and named them according to the chromosome structures. The Nramp protein sequence of kenaf was submitted to the SMART (http://smart.embl.de/, accessed on 17 July 2024) database as a search query sequence to verify the existence of the Nramp domain [44].

The candidate protein sequence files of kenaf were submitted to NCBI Batch CDD (https://www.ncbi.nlm.nih.gov/Structure/bwrpsb/bwrpsb.cgi, accessed on 31 May 2024) to obtain the conservation domain. We used the protein molecular weight analysis website ExPASy (http://web.expasy.org/protparam/, accessed on 1 June 2024) to analyze the physical and chemical properties of kenaf Nramp gene family proteins [24]. We used the WoLF PSORT website (https://wolfpsort.hgc.jp/, accessed on 31 May 2024) to determine the subcellular location of the family members [44]. Through the online website SOPMA (https:npsa-prabi.ibcp.fr/cgi-bin/npsa_automat.pl?Page=/NPSA/npsa_sopma.html, accessed on 10 May 2024), we predicted the secondary structures of the family proteins.

### 4.2. Sequence Alignment, Phylogenetics, Gene Structure, and Conserved Motif Analysis of Nramp Genes

To study the phylogenetic relationships of kenaf, *A. thaliana*, and tomato, the protein full-length sequences of plants were compared using the MUSCLE program provided by MEGA11.0 software [45], using the neighbor-joining (NJ) method with 1000 bootstrap replicates to build a multi-species phylogenetic tree [24,46,47]. The phylogenetic tree used Evolview v3 online (https://www.evolgenius.info/evolview/#/, accessed on 27 June 2024) software for visual analysis and beautification [24,36].

The gene structure was analyzed by Tbtools software. Conserved domains and motifs of the HcNramp proteins were analyzed with the Pfam 37.0 tool (http://pfam.xfam.org/,accessed on 17 July 2024) and the MEME Suite 5.5.5 (https://meme-suite.org/me/me/tools/meme, accessed on 14 May 2024), respectively. The relevant parameters were set as a maximum base ordinal number of 20, and the others used the default parameters. TBtools software [42,43] was used to visualize the phylogenetic trees and conserved motifs.

### 4.3. Cis Element Analysis of HcNramp Genes 

The ATG sequence of the genomic start codon was extracted from the *HcNramp* gene family as the promoter sequence, and then we used the online software of PlantCare (http://bioinformatics.psb.ugent.be/webtools/plantcare/html/, accessed on 30 May 2024) to analyze the promoter sequence [24,47]. The target cis-acting elements were screened in Excel, and then drawn with TBtools software [42,43].

### 4.4. Chromosome Localization and Collinearity Analyses of the Kenaf Nramp Gene Family

We extracted the position information of the *HcNramp* sequence from the kenaf GFF file and used TBtools software [42,43] to map the position distribution on the kenaf chromosome.

To explore the collinearity of *Nramp* genes in kenaf, *A. thaliana*, and tomato, whole-genome information was analyzed using TBtools software [42,43,48].

### 4.5. Plant Materials and Treatments

The kenaf “H368” variety was used as the test material, provided by the Institute of Bast Fiber Crops, Chinese Academy of Agricultural Sciences. Seeds were surface-sterilized with 75% alcohol for 2 min, washed with deionized water three times, germinated for one week, then transplanted into Hoagland nutrient solution for another week. Following this, they were cultured in 0/30 μmol/L of CdCl_2_ for two weeks. Subsequently, the plants were sprayed with 100 μmol/L of melatonin solution once every two days for a total of three times over a period of one week. The culture conditions were 25 °C/20 °C for 16 h/8 h (light/dark), relative humidity of 65%, and illumination of 12,000 lx. Finally, the roots, stems, and leaves (the third leaf from the top) of kenaf seedlings were sampled with liquid nitrogen and put in a refrigerator at −80 °C for further experiments. There were four treatments: control treatment (CK + water, CK-H), melatonin treatment (CK + melatonin, CK-M), Cd treatment (30 μmol/L CdCl_2_ + water, Cd-H), and Cd + melatonin treatment (30 μmol/L CdCl_2_ + melatonin, Cd-M). Nutrients were renewed every three days. There were three replicates for each treatment.

### 4.6. RNA Extraction and qRT-PCR Analysis

The total RNA was extracted from the root, stem, and leaf samples of kenaf by using the EASYspin Plus plant RNA rapid extraction kit. The NanoReadyF-1100 was used to determine the quality and concentration of RNA samples. Then, the first-strand cDNA was synthesized by using 5 × Prime Script RT Master Mix (TaKaRa). Taking the *Histone3* of kenaf as the reference gene [49], the primer sequence of *HcNramp* was designed by using TBtools and listed in Table S8. The qRT-PCR reaction was detected in the Step OnePlus Real-Time PCR System. The total reaction volume of PCR was 10 µL, including 1 µL of diluted cDNA, 5 µL of TB Green Premix Ex Taq (Tli RNaseH Plus) (2×) reagent, 0.2 µL of ROX Reference Dye, 0.2 µL of forward primer, 0.2 µL of reverse primer, and 3.4 µL of RNA-free H_2_O. The amplification conditions were as follows: in the first stage, 95 °C acted for 30 s; in the second stage, 95 °C for 5 s and 60 °C for 30 min, with 40 cycles; in the third stage, 95 °C for 15 s and 60 °C for 1 min. The expression of *HcNramp* genes was calculated using the 2^−△△ct^ method. Three replicates were set for each reaction.

### 4.7. Statistical Analyses

GraphPad Prism was used for statistical analyses. Error bars indicate mean ± SD and asterisks indicate statistical differences (n = 3; * *p* < 0.05, ** *p* < 0.01, and *** *p* < 0.001; ns, not significant; Student’s *t*-test) [50].

## 5. Conclusions

A comprehensive analysis of the *Nramp* gene family in kenaf was carried out in this study. Fifteen *HcNramp* genes were identified and further classified into three main phylogenetic subfamilies. A phylogenetic comparison of *Nramp* genes among kenaf, *A. thaliana*, and tomato was constructed, which provided some clues for understanding the evolution of *HcNramp* genes. All proteins of the HcNramp family were located in the plasma membrane, and they had highly similar exon–intron structures, motifs, and promoter elements in the same subfamilies. The distribution of chromosomes was not uniform, and the expression levels of different tissues in different treatments were also different. Most *HcNramp* genes were preferentially expressed in roots. These findings provide necessary information for understanding the potential role of the *HcNramp* genes in Cd stress and are also of great significance for screening Cd-tolerant genes in kenaf.

## Data Availability

All datasets generated for this study are included in the article/Supplementary Materials.

## Supplementary Materials

The following supporting information can be downloaded at: https://www.mdpi.com/article/10.3390/plants13172514/s1. Table S1: 15 protein sequences of HcNramp identified from kenaf. Table S2: Physical and chemical properties of *HcNramp* gene family members. Table S3: Secondary structure of HcNramp family protein. Table S4: List of 29 Nramp protein sequences. Table S5: List of the putative motifs of HcNramp proteins. Table S6: Collinearity information. Table S7: Transcriptome data of 15 *HcNramp* genes. Table S8: Primer sequences for ten target genes.
